# From fragmentation toward integration: a preliminary study of a new long-term care policy in a fast-aging country

**DOI:** 10.1186/s12877-019-1172-5

**Published:** 2019-06-07

**Authors:** Tzu-Ying Chiu, Hsiao-Wei Yu, Rei Goto, Wen-Lin Lai, Hsi-Chang Li, En-Tien Tsai, Ya-Mei Chen

**Affiliations:** 1 Graduate Institute of Long-term Care, Tzu Chi University of Science and Technology, Hualien, Taiwan; 2 Department of Gerontology and Health Care Management, Chang Gung University of Science and Technology, Taoyuan, Taiwan; 3 Graduate School of Business Administration, Keio University, Tokyo, Japan; 4 National Health Insurance Administration-Southern division, Ministry of Health and Welfare, Tainan, Taiwan; 5 Quixotic Implement Foundation, Nantou, Taiwan; 6 Shuan Lien Social Welfare Foundation, Taipei, Taiwan; 7 Institute of Health Policy and Management, National Taiwan University, No.17, Xu-Zhou Road, Taipei, 100 Taiwan

**Keywords:** Long-term care (LTC) plan 2.0, Tier ABC team, Integration mechanisms

## Abstract

**Background:**

Taiwan, one of the fastest-aging countries in the world, started implementing version 1.0 of its long-term care (LTC) plan in 2008. In 2017, LTC Plan 2.0 began a new era with its goal to integrate Taiwan’s fragmented LTC service system. LTC Plan 2.0 also aims to establish an integrated community-based LTC system incorporating both health care and disability prevention. This three-tier model consists of the following: two LTC services with a day-care center as their base and case management (Tier A), a day-care center and a single LTC service (Tier B), and LTC stations that provide primary prevention services and respite services for frail community-dwelling older adults to prevent further disabilities (Tier C). A defined cluster of agencies in a local area works together as a Tier ABC team. LTC Plan 2.0 is a new policy for Taiwan, and hence it is important to understand the agencies’ initial difficulties with implementation and identify future challenges to help further policy development.

**Methods:**

This preliminary study explored the challenges to implementing LTC 2.0 through in-depth interviews based on Evashwick’s integration mechanisms with representatives from three service teams. We interviewed three chief executive officers and three case managers.

**Results:**

We found that the LTC Plan 2.0 mechanisms for service integration have been insufficiently implemented. Recommendations include (1) Build up the trust between agencies and government, avoid duplication of LTC services within Tier ABC team, and encourage agencies within a team to create a shared administrative system with the same mission and vision. (2) Clarify the roles and responsibilities of government care managers and agency case managers. (3) Provide an integrated information system and create an official platform for sharing client records across different agencies and caregivers. (4) Establish a tool and platform to track the budget and payment across different levels of service as soon as possible.

**Conclusion:**

There is an increased demand for LTC services in Taiwan because of its rapidly aging population. Our findings shed some light on the challenges to developing integrated LTC services and thus may help both policymakers and service providers find ways to overcome these challenges.

## Background

Taiwan is one of the fastest aging countries in the world: it is estimated that the proportion of the population made up of people older than 65 years will increase from 10.7% in 2010 to 38.6% in 2060 [1]. In response, the government of Taiwan started its first long-term care (LTC) plan, version 1.0, in 2008 [2]. In 2017, Taiwan’s LTC plan version 2.0 (Plan 2.0) began a new era with its goal of integrating Taiwan’s fragmented LTC service system.

The LTC Plan 2.0 aims to establish an integrated community-based LTC system that includes both health care and disability prevention care [3]. In addition to the case management provided in LTC Plan 1.0, LTC Plan 2.0 established a new 3-tier service system to promote the use of integrated and coordinated LTC services. This 3-tier system includes the following types of LTC agencies, which provided tiered LTC services: Tier A—general service centers, Tier B—professional care centers, and Tier C—local service stations. The mission for each tier is described as follows: Tier A agencies coordinate LTC resources, especially services provided by Tier B and C agencies, and has to provide adult day care services as well. Tier B agencies aim to provide multiple professional care related services to meet specific LTC needs (e.g., home care, physical therapy). Tier C agencies provide hyperlocal, community-based drop-in services for community-dwellings for older adults who are frail or have light disabilities, with the goal of preventing further disabilities [3, 4]. An LTC service team includes 1 Tier A agency and several Tier B and C agencies. There were 9 LTC service teams certified by the Ministry of Health and Welfare in 2017 [3, 4]. In LTC Plan 2.0, a cluster of local agencies works together as a Tier ABC team. Each Tier A agency coordinates the LTC services provided by its team’s Tier B and Tier C agencies. The deputy ministers of the Ministry of Health and Welfare in Taiwan have said that the aim is to provide a Tier A agency in every township and a Tier B agency in every junior school district. The way of divide townships and districts was similar to other countries [5, 6]. In the next three to 4 years, the government in Taiwan has said it will establish 469 Tier A agencies, 829 Tier B agencies, and 2529 Tier C agencies [7].

The literature includes many reports of the benefits of mechanisms for LTC integration [8–12]. Evashwick [13] summarized four integration mechanisms: (1) Inter-entity organization and management, in which organizations cooperate with each other as a network; these organizations also have to share values, vision, mission, and culture [12, 13]. (2) Coordination of care, in which organizations focus on meeting clients’ needs and reducing variances of care [13]. (3) Integrated information systems, which allow client information to be shared between service providers, case managers, and family caregivers; integrated information systems also lead to more effective allocation of services [13, 14]. (4) Integrated financing, which expects to link all the possible funding sources as funding pool, and create a flexible funding to support clients’ social and medical needs in the continue care [13, 14].

The integration mechanisms (Inter-entity organization and management, Coordination of care, Integrated information systems and Integrated financing) that LTC Plan 2.0 is based on is new to Taiwan, and it will be important for policymakers both to understand current difficulties with plan implementation and to identify the challenges for LTC Plan 2.0 agencies. Therefore, our preliminary study aimed to use the integration mechanisms (Inter-entity organization and management, Coordination of care, Integrated information systems and Integrated financing) as a framework to examined experiences and challenges of integration faced by the LTC plan 2.0 agencies in Taiwan.

## Methods

This qualitative study used individual face-to-face interviews to examine the difficulties and challenges of integration when agencies implement LTC Plan 2.0.

### Sample

Taiwan began implementing LTC Plan 2.0 on January 1, 2017, In early 2017, 9 agencies in 20 counties were in the pilot stage of LTC Plan 2.0’s community-based integrated LTC services. From January 12 to Febrsuary 17, 2017, we interviewed three LTC Plan 2.0 Tier A agencies in north, west, and central Taiwan; interviewees included chief executive officers (CEO) or executive directors (ED) and case managers. The CEO and ED were responsible for planning and supervised the whole project, and the title of CEO and ED depended on the LTC agencies, but their duties of implementing and providing integrated LTC services were the same. The case managers coordinate and link LTC service resources under the supervision of the CEO or ED whose vision of providing integrated LTC services was carried out by case managers. They were also responsible for supervising case managers’ services quality. These two roles are inseparable in a team, and both of their perspectives regarding agencies’ experiences and changes in providing integrated LTC services under the new LTC Plan 2.0 were equally important. The Tier A agencies represented in the study interviews were certified as LTC Plan 2.0 Tier A by the Ministry of Health and Welfare in Taiwan and had the capacity to help establish Tier B and Tier C agencies.

### Semi-structured interviews

Each interview was conducted in person by two trained researchers, using semi-open questions. The interview guidelines were focused on the four integration mechanisms for system integration: (1) inter-entity organization and management: the study team examined how the agencies and government departments cooperate, and the difficulties they face as they work with different agencies. Do they have a regular meeting and can they set both short and long-term goals together? Besides, do they share the values, vision, mission, and culture of the agencies? Finally, can the agencies achieve consensus? (2) Coordination of care: how the agencies focus on meeting the clients’ needs and reducing variances of care and how to arrange the roles between government care managers and Tier A agency case managers. (3) Integrated information systems: How to share the client’s information among LTC service providers, case managers, and family caregivers, and enhance the better allocation of LTC services. (4) Integrated financing: To examine the present finance mechanism, and find out if the agencies link all the possible funding sources as a funding pool, and create flexible funding to support clients’ social and medical needs in the continue care [13]. The research team would inquire into the present situation of these four mechanisms, and based on them develop semi-structured interview questions regarding the experiences and challenges of integration faced by long-term care service teams (see Table 1 for interview questions).

**Table 1:** Interview guidelines

Mechanisms / Themes	Questions
1. Inter-entity organization and management	(1) How to work with different local government departments, which support medical care and social care?
	(2) How to arrange cases, how to assign cases among the tier ABC? And how to allocate the overall service hours among the tier ABC?
	(3) LTC Plan 2.0 emphasizes the integration of resources. What are the most challenging difficulties for the agency so far? (e.g., Agency role, Human resources, Service delivery, Coordination of different departments and agencies, Resource development), and any strategy to deal with the challenges?
2. Coordination of care	(1) How to coordinate recipients’ needs between Tier A and (government) care managers?
	(2) What are the difficulties between Tier A and (government) care managers when doing case management?
	(3) Any difficulty in developing service plans?
	(4) Any difficulty in implementing service plans, especially when services are provided by different agencies (e.g., gate-keeping, caseload)
3. Integrated information systems	(1) How to pass clients’ personal information and service plans among Tier ABC teams?
	(2) Possible solutions
4. Integrated financing	(1) What is the present payment mechanism?
	(2) What was the financial impact for the agency when the LTC Plan 2.0 policies started?
	(3) What are the difficulties for integrated financing?

### Data analysis

We used content analysis to analyze the interview transcripts, following these steps [15]:

1. Read the transcript over and over again.
2. Make initial codes when finding a relationship in the transcript.
3. List all the information codes and group them into themes and subthemes.
4. Name the themes and make sure each theme is distinguished from the others. Review all themes and codes to ensure that the themes cannot be merged.
5. Provide detailed descriptions of the data and elaborate the themes.

This study was approved by National Taiwan University Hospital Research Ethics Committee (Reference Number: 201706006RIND).

## Results

Three Tier A LTC service agencies were represented by individual interviews with 6 staff members. Agency and interviewee details are shown in Table 2. Table 3 summarizes the themes and subthemes found in the transcripts.

**Table 2:** Interviews and interviewees

Agency Code	Date of Interview	Interviewees	Agency Team Capacity
I	20170112	Chief executive officer Case manager	1A, 3B, 3C
II	20170123	Executive director Case manager	1A, 3B, 3C
III	20170216	Chief executive officer Case manager	1A, 2B, 4C

**Table 3:** Themes, subthemes, and cite frequencies

Mechanisms / Themes	Subthemes	Keywords	Cite Frequency
1. Inter-entity organization and management	1-1. Lack of policy integration between government departments	• Role, Standpoint	12
		• Government Administrative	6
		• Process, Procedure	
	1-2. Lack of understanding of the new policy among service agencies	• Inter-organization, Department, Linkage	5
	1-3. Lack of integration of vision and scope of service among service agencies	• Service Agencies	14
2. Coordination of care	2-1. Barriers to coordination between government care manager and agency case managers 2-2. Agencies’ lack of understanding of care coordination	• Case Management、Service Plan Development	14
		• Link the Resources	
3. Integrated information systems	3-1. Challenges to information sharing among service agencies	• Multi-information, Mix-information, Data, medical record	6
4. Integrated financing	4-1. Uncertainty about costs of recruiting new clients 4-2. Uncertainty about finance mechanisms	• Payment, Charge	5 4
		• Cost	

### Inter-entity organization and management

Referring to the inter-entity organization and management in Tier ABC teams, we generalized to four subthemes: (1) Lack of policy integration between the government’s health and social departments; (2) Lack of understanding of the new policy among service agencies; (3) Lack of integration of vision and scope among service agencies (shown in Table 3).

#### Lack of policy integration between government departments

Local governments administer different parts of the LTC system through their health and social departments. We found that lack of policy integration between these two departments of the local government, as well as between the central (national) and local governments resulted in great confusion for the Tier A agencies, especially when agencies raised questions about policy management. The rigid and inflexible policy of LTC Plan 2.0 also resulted in limiting the development of service plans tailored to clients’ needs.

*“The key of the LTC Plan 2.0 policy successes is whether the government and local government can respond to our problems rapidly. For example, when we had problems, we asked the social and health departments, but they gave us different answers. What should I[agency] do now?” (Agency I)*

*“We hoped that we would be more flexible in service plan arrangements across the social and health departments.” (Agency III)*

#### Lack of understanding of the new policy among service agencies

Although workshops were held to explain how the different tiers of agencies should work, agencies still did not clearly understand their own roles and responsibilities within their Tier ABC team, especially how to collaborate with each other. This was particularly the case regarding Tier C agencies: unlike Tier A and Tier B agencies, which had already provided some LTC services to disabled people and their families, Tier C agencies were new to the LTC system in Taiwan. LTC service agencies had to spend more time familiarizing themselves not only to their own new role in the integrated Plan 2.0 policy but also to the new service provided by Tier C agencies. The Tier A agencies reported that they were willing to spend extra time to figure out how to understand and collaborate with their teams under the new policy regulations. However, the process seemed to take more time than expected:

*“Even though the government held the session to illustrate the process and implementation for building up the [Tier ABC team], the role was still not clear…such as how would B and C fit in…” (Agency II)*

*“The government revised the rules according to the present situation, but the roles for Tier B and C were still not clearly shown.” (Agency II)*

*“The LTC Service agencies were not sure about becoming formal LTC Plan 2.0 service agencies.” (Agency II)*

*“Every agency needs to figure out their own services and services criteria [in the new policy regulations], because not everyone knew that…” (Agency II)*

#### Lack of integration of vision and scope among service agencies

Before LTC Plan 2.0 began, each of the LTC service agencies provided services separately, based on their own agency’s mission, rules, and culture. To provide integrated services according to the LTC Plan 2.0 policies, all agencies first must build trust and develop a common vision and mission, as well as administrative procedures. There were four subthemes to this challenge: (1) lack of trust and shared procedures among service agencies, (2) uncertainty about potential competition for LTC cases among service agencies, (3) anxiety surrounding the uncertainty of integration, and (4) lack of a common goal and mission among service providers.

#### Lack of trust and shared procedures among service agencies

The move from a fragmented service system to a system intended to integrate LTC services confused all the agencies interviewed. In particular, interviewees reported confusion on how to build a collaborative service model (including administrative processes for referrals) among service agencies.

*“The LTC Plan 2.0 policy aim is to integrate different LTC agencies, but how do we deal with the inter-entity [service agencies] problems when we refer to the process of service provision and service design in varied agencies? In the past, different agencies had different service delivery methods or models.” (Agency I)*

*“I am not sure I could refer the case to another agency, because we are not familiar with the referral criteria and process in the present.” (Agency II)*

#### Uncertainty about potential competition for LTC cases

The capacities and sizes of the agencies we interviewed were different, and most agencies were worried about losing their own cases when other agencies joined the system. This was due to both size differences between the agencies and lack of trust among them.

*“According to every agency’s capacity and size difference, is there any possibility the agency will be colonized by another better integrated one?” (Agency I)*

*“When linking other agencies to us, can we trust each other? Is there any possibility that we might lose our cases to the others? That would be a question.”(Agency I and Agency II)*

#### Anxiety surrounding the uncertainty of integration

All of the agencies demonstrated lack of confidence in the LTC Plan 2.0 policy. In addition, most of them showed great anxiety around the uncertainty of integration.

*“Anyway, there were a lot of existing problems in the policies we haven’t fixed yet.” (Agency I and Agency III)*

#### Lack of a common goal and mission among service agencies

Lack of a common goal and mission, which resulted in difficulty building trust among agencies, is the fundamental challenge for agencies to work together in Tier ABC teams.

*“If there is no practical standard for cross-agency cooperation, it is easy to result in misunderstanding among the Tier ABC [team]. For example, we have our own Tier ABC [team] with the same system, [and] we might have integrated [goals and mission] already based on our organization culture within our organization, but when we link with other agencies, can we trust the service quality for each other?” (Agency I)*

### Coordination of care

The challenges for coordination of care included confusion of job responsibilities between government care managers and Tier A agency case managers. Because both government care managers and agency case managers can provide similar functions in coordinating services for clients, great confusion—and lack of trust—has resulted for these two professions. Because of the overlapping nature of the responsibilities, agency case managers also worried that government care managers might send them more cases than they could afford to provide services to.

#### Barriers to coordination between government and agency staff

In Taiwan, government care managers are responsible for multiple tasks related to accepting LTC clients into the system, as well as establishing care plans and monitoring resources and outcomes before LTC Plan 2.0 started. Based on LTC Plan 2.0, the government care managers would continue to initiate all cases in their area of responsibility, and then the newly established LTC Plan 2.0 agency case managers would assess clients’ needs and connect clients with home care or community-based care as needed. However, when facing complex cases, government care managers only make the “care plan”; they then assign and refer these complex cases to the Tier A case managers, and these case managers assess client needs and link clients to necessary care services. Government care managers (who conduct evaluations and provide care plans) and Tier A agency case managers (who provide service plans) were not clear about which role was supposed to take responsibility for developing evaluations, care plans, and service plans. In addition, they also showed a lack of trust toward each other.

*“Do [government] care managers have enough time and capacity to evaluate the client’s need based on such higher caseloads?” (Agency I)*

Agency case managers thought they were entitled to evaluate the client’s need and even to develop the care plan and service plan together with the government case manager, because they were supposed to know the care recipients best. However, they were worried that the government care manager might give them too many cases, because they might do better jobs.

*“Because our [agency] case managers were more engaged with the communities for a long time, they must be the people who know the recipients’ needs best. In contrast, if the [government] care managers share too much work with the case managers, they [the case managers) will get too much caseload. Therefore, how to arrange the work for these two kinds of staffs needs to be considered.” (Agency I)*

#### Agencies’ lack of understanding of care coordination

Heavy caseloads resulted in Tier A case managers trying to shift caseloads to Tier B agencies, which are agencies that provide only a single service. This resulted in yet more confusion in care coordination across different coordinating professions.

*“We thought the agency case manager [role could be] not only for Tier A but also for Tier B, because Tier B has the capacity to do service plans in their area of responsibility. Then the case manager in Tier A just would review and check the procedure. In other words, transferring of agency’s case manager’s rights form Tier A to Tier B can reduce the case managers’ loads in Tier A. Tier B [staff] can make the client’s service plan more appropriate.” (Agency I)*

### Integrated information systems

Collaborating on integration of information systems was another challenge for LTC Plan 2.0 agencies. Because there was no integrated information system available, most of our interviewees pointed to two challenges needing solutions: (1) Delivery of client information across a Tier ABC team was not smooth. (2) There was no mechanism to monitor how different agencies delivered services to clients. One concern was that clients might use either more or less services than planned by their agency case managers, while receiving multiple LTC services from different agencies.

#### Challenges to information sharing among service agencies

Due to differing administrative processes, service agencies had different referral processes, which resulted in difficulties when passing client information across service agencies within Tier ABC teams.

*“There is no information system to pass the client across different service providers.” (Agency I)*

Since there are currently no existing information systems or platforms on which different agencies can report the amount of services and care provided to a client, case managers in Tier A agencies were concerned about how to monitor the services and care provided by other agencies.

*“If the clients receive the multi-LTC services from different service providers, but there is no electronic system to do the overall hour control, now our staffs need to record every client’s status. These kinds of processes will take time and [it is] easy to make mistakes.” (Agency I)*

### Integrated financing

The participants mentioned that further challenges were found in the fourth theme: integrated financing. Almost all participants reported that the payment mechanisms across different tiers of agencies were not clear. Interviewees from Tier A agencies were afraid that the services their agencies had provided would not be reimbursed by the government. They thus tended not to recruit new clients and develop new services, particularly services that were also provided by Tier C agencies.

#### Uncertainty about costs of recruiting new clients

The present LTC Plan 2.0 payment processes lack incentives. There is no extra payment for recruiting more clients. Thus, Tier A agencies focused on maintaining their current services for their original clients instead of recruiting new clients. In addition, agencies hesitated to recruit new clients because they worried that they would increase their agency’s costs without full knowledge of the payment scheme. Rumors about future possibilities for capitation also increased Tier A agencies anxiety about recruiting new cases.

*“In the beginning, we couldn’t receive too many cases, because the payment was uncertain—would the government recognize all the costs we’ve incurred? There is a doubt.” (Agency I)*

*“There is no standard payment for LTC Plan 2.0, so now we focus on how to cultivate Tier C and to develop a model which fits local communities’ needs…and not to recruit more clients.” (Agency II)*

*“How much money does Tier A need to pay for Tier C? How to settle it? There are no specific rules and definitions yet.” (Agency II)*

*“If the payment was capitated, the administrative cost in Tier A was much more, and then Tier B must be reducing the cost. Can Tier A accept such outcomes?” (Agency I)*

## Discussion

All four key system integration mechanisms are important to integrating LTC services among Tier A, Tier B, and Tier C agencies in Taiwan. During the first year of implementing the LTC Plan 2.0 policies of integrated LTC services, most challenges were seen. In general, when we explored the experiences of Tier A agencies with the current Taiwan LTC Plan 2.0, we found that there is room for improvement.

### Inter-entity organization and management

For inter-entity organization and management, we found that there were no clearly defined roles offered by governmental health and social departments when service agencies communicated problems or questions.

#### Integrating government departments responsible for long-term care

In Taiwan, a local government’s health department and social department are responsible for administrating different types of LTC services. Our findings show that service agencies have struggled with having two local government departments co-supervising the development of Tier ABC team services in the local area. Between the two departments, the main roles and responsibilities for integrated services are not clearly identified, and this confusion has created a gray area about how LTC agencies are to practice the Plan 2.0 policy. In 2017, a few local governments, such as New Taipei City and Taichung City, were trying to solve this problem by merging their social and health departments [16]. We expect that this kind of governmental integration process will facilitate the integration of LTC services. In addition, when only a single governmental department is responsible for coordinating LTC, clients and their families will have a single access point to apply for services [3, 17].

#### Build up the trust, and sharing a common administrative system and vision

Our study also found that most agencies were not familiar with how to build a collaborative service model among service agencies. Agencies were not clear about their roles and responsibilities. Due to size differences, agencies were afraid they would be competing for the same target clients.

Past literature has suggested that when building integrated LTC systems, it is important to put the integration mechanisms and organizational structure in place first [13]. Duplication of services provided by different agencies should be avoided. In addition to avoiding overlap in service areas, the target client population also should not overlap [13]. At the beginning of implementation of Taiwan’s LTC Plan 2.0, we found that Tier A and Tier B agencies could provide a range of LTC services, but both were required to provide a common service: day care center. In other words, although Tier A and Tier B agencies, by policy, collaborated in their Tier ABC team, they were actually competing for the same target clients in their local area, and most of the interviewees in this study were worried that they would lose cases.

In addition, Griffith [18] has suggested that a common mission and vision is key to integrating organizations with common short-term and long-term goals. In our study, we found that service agencies that shared the same administrative system and had a shared common mission and vision seemed to have adjusted better to the new policy. These agencies already knew what they were doing and how to work with each other in terms of adding the new integration tasks to their own LTC Plan 2.0 responsibilities. In contrast, agencies without a common administrative system and common vision suffered from trying to integrate. Therefore, building up agencies’ common mission and vision for the integration mechanisms is essential [10, 19, 20]. From the perspective of LTC Plan 2.0 policy developments, agencies that are from the same administrative system and with the same value, culture, mission, and vision may find it easier to integrate their services.

Heenan [21] mentions that well-developed communication strategies can provide strong support for agencies and clinicians working together and can reduce misunderstanding among different agencies. In addition, clinicians will be more at ease when they share the same goals and priorities. The literature has shown that it is helpful to provide regular meetings and work-based training and education programs to build up consensus among different organizations [21, 22]. A horizontal integration might serve this purpose [13].

Coordination through the same administrative system may ease agencies’ concern over losing cases to other agencies. However, in the current LTC Plan 2.0 policies, governments do not encourage the agencies in Tier ABC teams to come from the same administrative system. Governments and policy makers might be afraid that LTC services will be monopolized if all service agencies in a team are from the same agency system [3]. Unlike in Japan, each service agency in Taiwan has a case limit. Agencies have to share cases with other agencies when case loading reaching ceiling [23]. Therefore, current policy appears to limit the integration of the LTC system in Taiwan.

### Coordination of care

For coordination of care, the main challenges for agencies were confusion over which role—between government care manager and Tier A agency case manager—was supposed to take on the responsibility of developing a client’s care or service plan.

In the Plan 2.0 LTC system, coordination of care is key to success. Some care management problems existing since the beginning of LTC 1.0, such as service fragmentation, have not been fixed yet. In Taiwan, government care managers are responsible for multiple tasks, including reviewing applications for LTC services, checking qualifications, interviewing the client, making an evaluation and care plan, coordinating resources, and monitoring the client’s LTC services provided.

Given that the number of LTC clients keeps increasing, it’s not easy for government care managers to cover all these tasks. According to regulations, the ideal caseloads are 150 to 200 clients to a care manager, but average actual caseloads were around 500 clients [3, 24]. With such high caseloads, care quality will decrease [25]. As we saw in our interviews, LTC Plan 2.0 is trying to overcome this challenge by creating the new role of Tier A agency case manager. The initial idea for this agency case manager role was to help integrate services among a Tier ABC team and to share in government care managers’ caseloads. The common goal of these two roles—government care managers and agency case managers—was to find the resources to meet clients’ needs in the community.

However, the government did not clearly define the differences in function and content between these two clinical care coordinators [26]. Therefore, the roles of government care managers and agency case managers in the LTC system varies in every county. Some counties even have both care manager and case manager visit each client together, despite the LTC Plan 2.0 strategies aiming to support already limited LTC staff.

Good care coordination is known to improve care quality, allocate the resource as gatekeeper, and save money [27, 28]. Therefore, the division of responsibilities among clinical workers is a high-priority problem for the Taiwanese government to solve.

### Integrated information systems

For the integrated information systems, the problem was the lack of a client’s information transfer platform across agencies, service providers, and caregivers.

To achieve a comprehensive integrated LTC system, information integration is an important mechanisms [13]. Our present study found that the lack of a secured integrated information system resulted in inefficiency between agencies. Without such a system, clinicians within the LTC service agencies need to spend a lot of time on paperwork, which results in lower efficiency in both administrative processes and service provision [3]. The literature has proven that an integrated information system can provide simple access to client data, which can be sent across multiple sites and providers. Such a standardized system makes service provision more efficient and produces better outcomes [13, 14, 29]. Electronic records also allow the system more functionality, such as creating clinical guidelines, establishing quality control, evaluating improvement of quality indicators, and monitoring clinical outcomes [13, 14, 29]. The key is that such information sharing can facilitate and support both external (accounting) and internal management (decision making) functions of LTC service agencies [14].

Taiwanese governments, both local and central, are aware of the problem of not having an integrated information system. However, building an integrated information system was not on the priority list for LTC Plan 2.0. Our study findings may encourage the government to rethink this priority. Intergration of information system may also be easier to solve than some other integration mechanism given that Taiwan has expertise in IT development. Now the government in Taiwan needs to consider how to link and coordinate different data sources setting in different government departments. With an integrated information system, LTC service agencies will able to see the whole picture for their clients and provide better care. Creating integrated information systems with electronic records and a well-designed integrated financial mechanism across different service providers seemed to be a minor challenge that can be resolved quickly.

### Integrated financing

For the integrate finance, the lack of tools to track the budget and payment across different levels of service also served as a barrier to increasing caseloads and providing integrated services.

Financial payment mechanisms are fundamental to the LTC service industry, and our findings clearly show that all of the agencies we interviewed were struggling with the current payment mechanism. In addition, lack of tool to track the budget and payment across different levels of service also served as barrier to increasing caseloads and providing integrated services. This resulted in hesitation to recruit new clients and provide new services in order to avoid potential lost costs. In the past, LTC payments in Taiwan were fee-for-service, which resulted in fragmented services [3, 30]. Many LTC management guidelines recommend that capitation, or package payments per user, is the best way to reduce the waste in duplicated services and to allocate resources more efficiently [14, 17]. In early 2018, the government of Taiwan did revise the payment mechanism. Today it is moving in the right direction even though its effectiveness is still unknown. Further Studies is needed on how to integrate the LTC payments in Taiwan and move the system from being a fee-for-service model to a packaged payment system.

The government might also think about ways to help agencies understand the payment scheme. All agencies are nonprofit, and all had concerns about not being able to sustain themselves, especially when the policy changes so fast. Agencies’ fear of further policy changes and that they would not be able to make their budgets balance made them reluctant to take new cases.

Following a new policy change in January of 2018, Tier ABC teams are no longer bound to each other [31]. Now, most agencies are planning to become Tier A agencies and have their own case managers. It will be of great interest in the future to evaluate whether this policy change will provide more flexibility for integrating LTC services or whether services will become more fragmented given that every agency wants to “manage” clients themselves.

### Limitation

To provide better LTC services, the government in Taiwan is continually revising LTC Plan 2.0 regulations based on the problems encountered by LTC service providers. Therefore, some problems encountered in interviews and mentioned in this manuscript may already have been solved [32]. Although we only interviewed three teams, we found information reached saturation by the time we finalized with the third agency. Since our study was conducted only several months after the new LTC Plan 2.0 policy was launched, at that time, there were just nine agencies chosen by the Ministry of Health and Welfare to serve as the pioneer in the provision of integrated LTC services in Taiwan and we interviewed one-third of these agencies. Also, there were only one CEO or ED and two case managers for each team in the LTC Plan 2.0 regulation, we have included all team members in our interviews. Their feedback together provided a more complete picture regarding the challenges of providing integrated LTC service in Taiwan. Although the sample size may seem small, our research acts as a preliminary study, and its findings may serve as a foundation for future research studies. The findings also act as a reference for other service teams who are interested in becoming a Tier ABC team and provide integrated LTC services.

## Conclusion

Our study findings shed light on outstanding needs both for Taiwan and for the many countries which are about to develop integrated LTC services. In summary, we find that the mechanisms of integration have not been fully implemented. Although service agencies suggested that coordination of care is key to success in providing integrated LTC services, our findings suggested that the following recommendation are importance as well. (1) Build up the trust between agencies and government, avoid duplication of LTC services within a local Tier ABC team, and encourage agencies within a team to create a shared administrative system with the same mission and vision. (2) Clarify the roles and responsibilities of government care managers and agency case managers. (3) Provide an integrated information system and create an official platform for sharing client records across different agencies and caregivers. (4) Establish a tool and platform to track the budget and payment across different levels of service as soon as possible.

In conclusion, the need for LTC services in Taiwan is increasing due to a rapidly aging population; our findings shed some light on ways that both governments and service providers can overcome future challenges to developing an integrated LTC service system.

## Data Availability

The datasets generated and analyzed during the current study are not publicly available due to the data form the LTC agencies interview but are available from the corresponding author and LTC agencies on reasonable request.

## Abbreviations

LTC Plan 2.0: Long-Term Care Plan version 2.0
LTC: long-term care
